# Unpacking the blood flow restriction device features literature: autoregulation

**DOI:** 10.3389/fspor.2024.1455277

**Published:** 2024-10-24

**Authors:** Nicholas Rolnick

**Affiliations:** ^1^ Department of Exercise Science and Recreation, CUNY Lehman College, New York, NY, United States; ^2^ The Human Performance Mechanic, New York, NY, United States; ^3^ The BFR PROS, New York, NY, United States

**Keywords:** Delfi, autoregulation, resistance training, limb occlusion pressure, arterial occlusion pressure, BFR training

## Introduction

Blood flow restriction (BFR) exercise has gained popularity in various practice settings (1) due to its superior training benefits with low-intensity resistance (≤50% one-repetition maximum, 1RM) and aerobic exercise (≤50% VO_2_max), surpassing intensity-matched non-BFR exercise (2). BFR is typically performed using a specialized cuff applied to the proximal part of the limb, inflated to a percentage (40%–80%) of the total pressure needed to fully occlude arterial inflow at rest, known as limb occlusion pressure (LOP) (2).

However, as BFR has expanded into more diverse practice settings and more manufacturers are producing BFR cuffs for consumer purchase, device features such as the presence of autoregulation have added the potential for further heterogeneity in research based solely on the device features of the BFR cuff (3). As a product of this interest in BFR and the increased diversity of BFR products, the pace of rigorous research addressing these differences between devices is lagging.

Therefore, the aim of this opinion piece is to highlight two recently published studies that investigated autoregulation and discuss device-specific nuances such as the responsiveness of the autoregulation feature itself that don't necessarily make it into publications. The hope of highlighting these nuances is for researchers and clinicians implementing BFR to be better informed about the BFR stimulus imparted in their studies or their clients/patients.

## Autoregulation of applied BFR training pressures

Autoregulation refers to a BFR cuff's ability to adjust the set/interface pressure according to the phase of muscular contraction (3). For example, when the exerciser is lifting a weight and the muscle is undergoing a shortening contraction (e.g., the concentric portion), the cuff with autoregulation enabled is supposed to quickly adjust and reduce the applied BFR pressure to the limb to maintain a relatively consistent set/interface pressure (±15 mmHg) (4). Conversely, when the muscle is lengthening, there is not the same amount of pressure applied from the limb to the cuff, so the cuff is supposed to quickly adjust by pumping more air into the cuff to compensate. However, the responsiveness of this adjustment varies significantly between devices that claim to offer autoregulation (based on 7 years of author experience with these devices). A common but flawed assumption is that all autoregulated cuffs provide a similar stimulus (e.g., acute exercise performance or perceptual responses) across different models. This assumption can impact our understanding of autoregulation's role in BFR training if not considered in research methods or exercise prescriptions.

As of mid-2024, there exists only four published studies that have considered the autoregulation feature of a BFR cuff in the methodological design (5–8). However, only two of them (7, 8) are able to provide insights into the causative impact of autoregulation on exercise performance and perceptual responses that can inform its utility. Isolating the impact of autoregulation can *only* be done by implementing methodologies that perform BFR exercise with a cuff of the same material, make, and width but allow for intra-exercise regulation of applied BFR training pressures or not. Any other methodology where the cuffs are not identical cannot inform us of the potential impact of a cuff's autoregulation capabilities if other cuff-related factors are present. Thus, different BFR cuffs capable of autoregulation can be more effectively investigated when the only variable that differs between conditions is the presence/absence of autoregulation.

Important for this discussion is that both of the studies previously mentioned (7, 8) utilized two different BFR cuffs and had at least one condition of the experiment performing 4 sets of exercise to volitional failure (Table 1).

**Table 1:** Comparisons of the relevant study characteristics between Jacobs et al. (7) and Rolnick et al. (8).

	Jacobs et al. (7)	Rolnick et al. (8)
Participants	56 physically active adults (31M, 25F); 27.7 ± 9.7 years old	20 physically active adults (13M, 7F); 23 ± 5 years old
Protocol	4 sets of unilateral leg extensions (90˚ flexion to extension range of motion) to volitional failure performed at ∼20% 1RM with 4 scheme with 45 s inter-set rest and 2 s concentric/eccentric tempo.	4 sets of bilateral dumbbell wall squat exercise (90˚ flexion to extension range of motion) performed at ∼20% 1RM to volitional failure with 60 s of inter-set rest and 2 s concentric/eccentric tempo.
BFR Device	SmartCuffs Generation 3 PRO (10.14 cm width)	Delfi Personalized Tourniquet Device (11.5 cm width)
BFR Pressures/Application	60% LOP determined in sitting (average applied pressure: ∼131 mmHg) applied continuously and deflated after set 4.	60% LOP determined in supine (average applied pressure: 113 [left leg]-123 [right leg] mmHg) applied continuously and deflated after set 4.
Repetitions Performed	AUTO achieved 29.6% increase in total reps over NAUTO (199 vs. 161).	No differences in total repetitions performed between AUTO and NAUTO (53 vs. 52).
Perceptual Responses	Statistically lower RPE in AUTO compared to NAUTO (17.2 vs. 17.7; classified as “very hard” exertion) Statistically lower RPD in AUTO compared to NAUTO (8.1 vs. 8.5; classified as “very severe” discomfort)	No differences in RPE between AUTO compared to NAUTO (8.2 vs. 8.5 on 1-10 Borg RPE scale; classified as “really hard”) No differences in RPD between AUTO and NAUTO (6.2 vs. 6.6; classified as “high” discomfort)
Author Conclusions	AUTO allows for greater exercise performance compared to NAUTO with less RPE/RPD.	The presence of AUTO has negligible impact on exercise performance and perceptual responses compared to NAUTO.

AUTO, autoregulated; NAUTO, unregulated; BFR, blood flow restriction; 1RM, one-repetition maximum; RPE, rating of perceived exertion; RPD, rating of perceived discomfort.

Jacobs et al. (7) utilized the Generation 3 SmartCuffs PRO (SmartTools, Ohio, USA) and had participants perform 4 exercise sessions each separated by at least a 7-day washout period. Each participant was randomized into a protocol that first performed a fixed 75-repetition (1 × 30, 3 × 15) scheme with or without autoregulation (e.g., the first two sessions) enabled followed by a repetition protocol of 4 sets of volitional failure in a similar randomized fashion (e.g., the last two sessions). They concluded that during 4 sets to volitional failure, the autoregulation-enabled cuff allowed participants to perform 29.6% more leg extension volume with less perceptual demands and delayed onset muscle soreness than the same exercise performed without autoregulation (7). Acute safety between the autoregulation and non-autoregulation conditions was assessed by the occurrence of adverse responses categorized in accordance with the classification proposed by Minniti et al. (9). When compared in totality, the autoregulation condition experienced a 3× risk reduction in the occurrence of minor adverse responses (4 vs. 12) (e.g., dizziness) across both repetition protocols compared to the non-autoregulated cuff condition. Most of these adverse responses occurred in the first two sessions (7 in non-autoregulated and 1 in autoregulated) where the fixed 75-repetition protocol was performed, potentially providing support for use of autoregulation to enhance the acute safety profile of BFR exercise during initial training exposures. However, the number of adverse responses noted in the last two sessions to volitional failure between conditions were similar (5 in non-autoregulated and 3 in autoregulated). Given the experimental methodology employed in Jacobs et al. (7) where participants performed the 75-repetition fixed protocol prior to exposure to the failure repetition scheme, I speculate that autoregulation employed during the initial fixed repetition training protocol mitigated the occurrence of minor adverse responses. However, once the participants were exposed to BFR, the presence of autoregulation had minimal influence on the occurrence of adverse events. If we take this study at face value, we can surmise that autoregulation should be implemented in practice—at least during the initial training sessions—because of positive effects it confers with resistance exercise and reduction in minor adverse responses in this study.

Conversely, Rolnick et al. (8) investigated similar outcomes related to acute exercise performance and perceptual responses, utilizing the Delfi Personalized Tourniquet device (Owens Recovery Science, San Antonio, TX). Their trial demonstrated that when autoregulation was enabled during 4 sets of bilateral wall squat exercises to volitional failure, no significant differences were found between autoregulation-enabled and non-autoregulation BFR, applied using the same cuff, in terms of performance (e.g., repetitions to volitional failure) or perceptual outcomes (e.g., ratings of perceived exertion and discomfort). Moreover, no adverse responses to BFR were observed in either arm of the trial. This highlights a case where a BFR device feature (e.g., autoregulation) produced differing acute outcomes across two distinct studies.

Practical hands-on experience with the autoregulation feature of both devices sheds light on the potential reason for the responses observed. The Delfi Personalized Tourniquet device is a retrofitted surgical tourniquet device with a microprocessor and air compressor capable of making quick adjustments of applied pressure during dynamic contractions (10). Its responsiveness is robust. Prior research has indicated that it can maintain set/interface pressure (±15 mmHg) consistently before, during and post-exercise compared to 5 other commercial or research-based BFR cuffs [e.g., Occlusion Cuff (The Occlusion Cuff LTD., Belfast, Ireland), SmartCuffs Gen 2 BFR cuff (non-autoregulated), Suji BFR (Suji, Scotland, UK), B-Strong (B-Strong Training Systems, Utah, USA), SAGA Fitness BFR Cuffs (Saga Fitness, Newstead, Australia)] (5, 6). However, the Delfi Personalized Tourniquet device is costly (>$5,000 USD), limiting widespread adoption. On the other hand, the SmartCuffs PRO is a smaller, more portable and affordable device (∼$1,000 USD), although significantly less studied. Due to the smaller size of the SmartCuffs PRO (Generation 3) and the motor powering the air flow, I speculate that the autoregulation function is not as robust as the Delfi Personalized Tourniquet device, leading to significant delays between inflation and deflation of the cuff as the exerciser is alternating between concentric and eccentric contractions. As a result of this difference in responsiveness, we can begin to formulate hypotheses about the divergent results observed between exercise performance and perceptual responses in the two studies.

It is of my strong opinion that the observations on exercise performance and perceptual demands in both studies can be explained by considering the responsiveness of each cuff's autoregulation feature. What likely occurred in Jacobs et al. (7) is that during the inter-repetition transition between concentric and eccentric phases, the set/interface pressure was significantly altered such that minimal pressure was applied to the limb in the eccentric phase, allowing blood flow to enter/escape the limb, reducing the BFR stimulus itself since it is predicated on partial arterial restriction and venous occlusion determined at rest (2). While no measures of intra-exercise blood flow were taken, the hypothesis of significant blood reperfusion is supported by the larger volume of exercise performed (29.6%) and the reduced perceptual demands compared to the non-autoregulated condition (7). Moreover, the incidence of adverse responses in both fixed and failure repetition schemes could similarly be explained by the autoregulated condition experiencing a greater degree of reperfusion per repetition, limiting the detrimental metabolic impact at the muscular level that may impact performance. The autoregulated condition may have provided a better maintenance of blood flow to the limb during exercise compared to the set pressure of the non-autoregulated cuff condition. As Rolnick et al. (8) utilized the Delfi Personalized Tourniquet device with a tighter responsiveness and better ability to maintain the applied pressure throughout the contraction duration (5, 6), they observed no discernible differences between BFR conditions on performance and perceptual outcomes. This can be explained by blood flow in the limb being contained in both conditions regardless of the presence of autoregulation. However, it cannot explain the absence of adverse responses in all conditions, warranting future research into what factors play the largest role in experiencing adverse responses to BFR exercise. Last, it is worth mentioning that the cuff width of the Delfi Personalized Tourniquet device is 13% greater (11.5 cm vs. 10.14 cm) than the SmartCuffs PRO which possibly could have impacted the observed responses in the two studies. However, prior research has shown that despite resting LOP values being different between cuffs of different sizes, the acute responses at rest (11) as well as perceptual, muscular, cardiovascular, and performance (e.g., repetitions to failure) to upper body BFR exercise appear similar (5 cm vs. 5.5 cm; 10%) across two different LOP prescriptions (40% and 80% LOP) (12).

It's important to highlight these studies because it informs us that the acute response to BFR exercise can be altered with a responsive autoregulation feature. Insofar as the limited body of evidence suggests, researchers (and clinicians using these cuffs) need to carefully consider whether the cuff employed has an autoregulation feature, and if so, its potential to induce a consistent BFR stimulus. When scrutinizing the literature, importance should be paid to fixed repetition scheme methodologies, as it appears that a less responsive autoregulation feature reduces the BFR stress per repetition (indicated by 29.6% more volume to exhaustion). Thus, the participants exercising with a BFR cuff that has a less responsive autoregulation feature are likely exercising further away from muscular failure, potentially leading to less effective muscular outcomes compared to a more responsive autoregulation feature.

## Conclusions and future directions in autoregulation

As research continues to be published exploring device features such as autoregulation, it is prudent to recognize that not all autoregulation enabled cuffs perform equally, as evidenced by the preliminary body of research (7, 8). BFR providers and researchers should consider that the responsiveness of the autoregulation feature has a significant impact on the overall acute physiological response. This may lead to potentially altering chronic outcomes when different cuffs capable of autoregulation are implemented in practice, although a recently published paper showed no differences in longitudinal outcomes between unregulated and autoregulated devices (*n* = 81 studies) (13).

Research should continue to explore the relevancy of the acute responses to BFR exercise with cuffs of different autoregulation responsiveness, as well as whether the differences in acute responsiveness between cuffs impacts longitudinal outcomes of interest such as improvements in muscle hypertrophy, strength or aerobic capacity. This is an area of high interest for the BFR community.
